# CRISPR for accelerating genetic gains in under-utilized crops of the drylands: Progress and prospects

**DOI:** 10.3389/fgene.2022.999207

**Published:** 2022-10-06

**Authors:** Kiran K. Sharma, Sudhakar Reddy Palakolanu, Joorie Bhattacharya, Aishwarya R. Shankhapal, Pooja Bhatnagar-Mathur

**Affiliations:** ^1^ Sustainable Agriculture Programme, The Energy and Resources Institute (TERI), India Habitat Center, New Delhi, India; ^2^ International Crops Research Institute for the Semi-Arid Tropics (ICRISAT), Patancheru, Hyderabad, India; ^3^ Department of Genetics, Osmania University, Hyderabad, Telangana, India; ^4^ Division of Plant and Crop Sciences, School of Biosciences, University of Nottingham, Nottingham, United Kingdom; ^5^ Plant Sciences and the Bioeconomy, Rothamsted Research, Harpenden, Hertfordshire, United Kingdom; ^6^ International Maize and Wheat Improvement Center (CIMMYT), México, United Kingdom

**Keywords:** CRiSPR/Cas, food security, gene editing, green revolution, new breeding technologies, speed breeding, underutilized crop, genetic gains

## Abstract

Technologies and innovations are critical for addressing the future food system needs where genetic resources are an essential component of the change process. Advanced breeding tools like “genome editing” are vital for modernizing crop breeding to provide game-changing solutions to some of the “must needed” traits in agriculture. CRISPR/Cas-based tools have been rapidly repurposed for editing applications based on their improved efficiency, specificity and reduced off-target effects. Additionally, precise gene-editing tools such as base editing, prime editing, and multiplexing provide precision in stacking of multiple traits in an elite variety, and facilitating specific and targeted crop improvement. This has helped in advancing research and delivery of products in a short time span, thereby enhancing the rate of genetic gains. A special focus has been on food security in the drylands through crops including millets, teff, fonio, quinoa, Bambara groundnut, pigeonpea and cassava. While these crops contribute significantly to the agricultural economy and resilience of the dryland, improvement of several traits including increased stress tolerance, nutritional value, and yields are urgently required. Although CRISPR has potential to deliver disruptive innovations, prioritization of traits should consider breeding product profiles and market segments for designing and accelerating delivery of locally adapted and preferred crop varieties for the drylands. In this context, the scope of regulatory environment has been stated, implying the dire impacts of unreasonable scrutiny of genome-edited plants on the evolution and progress of much-needed technological advances.

## Introduction

Climate change, population growth, pandemic, conflicts and rising socio-economic disparities are putting considerable pressure on the already stressed food systems. While the precise impacts of climate change are still unclear, unpredictable climate changes are expected to impact several vulnerable regions more adversely. This requires informed approaches to address sustainability issues to meet the future food needs. While staple crops have limited resilience to changing climate in the drylands, locally grown underutilized crops despite being vital for diverse nutrient and local adaptations are grown in low-input conditions. Underutilized crops (under-researched compared to staple crops; Chapman et al., 2022) have significant potential to aid food security through increased food production in challenging environments where major crops are severely limited (Mayes et al., 2012). More recently, the discovery of genomes and candidate genes have aided the study of underutilized cereal and legumes and provided syntenic comparisons for the evolution of C4 photosynthesis, with the potential to improve the photorespiration efficiency, drought tolerance, and nutritional traits. However, significant efforts are still needed to identify and understand the underlying allelic variation for breeding applications (Chapman et al., 2022).

Advanced breeding technologies such as genome editing hold immense potential for improving crop yields and quality by inducing precise genetic alterations in the targeted genomes (Wolt et al., 2016; Miladinovic et al., 2021). Emergence of programmable site directed nucleases (SDNs) such as zinc finger nucleases or ZFNs (Carroll, 2011), transcription activator-like effector nucleases or TALENs (Zhang et al., 2013), clustered regularly interspersed short palindromic repeats or CRISPRs (Jiang et al., 2013a), and more recent base editing and prime editing tools have provided technological breakthrough for inducing precise and rapid genetic variations in organisms including plants (Komor et al., 2016; Mishra et al., 2020; Xu et al., 2020; Miladinovic et al., 2021). SDNs cleave the DNA sequence at specific sites and repair the double strand breaks (DSBs) through homologous recombination (HR) or non-homologous end joining (NHEJ) repair pathways, resulting in sequence replacements or creating insertions or deletions (INDELs) at predefined sites. Several successful applications have been reported for trait improvements in plants such as poplar (Fan et al., 2015), soybean (Li et al., 2015; Bao et al., 2019), wheat (Cui et al., 2019), tomato (Nekrasov et al., 2017), sorghum (Li A. et al., 2018), cassava (Hummel et al., 2018) and rice (Endo et al., 2016) for addressing complex traits such as heterosis (Wang et al., 2019), nutrition (Ku and Ha, 2020), stress tolerance (Jain, 2015) and yields (Huang et al., 2018). A detailed analysis of the applications of CRISPR in crops of tropical origin for better adaptation to current environmental conditions and market needs has recently been made (Rojas-Vasquez and Gatica-Arias, 2020) including regulatory environment in Africa (Tripathi et al., 2022). This article reviews the significance of genome editing tools in general, and the evolving CRISPR system and its applications for creating new precision breeding opportunities for important subsistence crops of the drylands.

Genome editing tools use different mechanisms for the recognition of target DNA. For example, while ZFNs and TALENS use the DNA-protein interactions, the CRISPR/Cas relies on DNA-RNA interactions (Gaj et al., 2013). The first generation SDNs such as ZFNs were used for editing a range of plant genomes (Lloyd et al., 2005; Kumar et al., 2015; Petolino, 2015), high skills and cost hindered its widespread applications (Sanjana et al., 2012). TALENs emerged as a relatively easy tool that still required sound molecular biology skills for construct preparation (Boch et al., 2009; Boch and Bonas, 2010; Li et al., 2011). However, in the last decade, CRISPR/Cas9 tools have been most efficient and successfully used for vast range of applications due to their low cost, effectiveness, and user friendliness, thereby providing attractive options for precision plant breeding (Hilscher et al., 2017; Ma et al., 2018; Oliva et al., 2019).

CRISPR/Cas has not only advanced at a very fast pace but has been efficient in simultaneous editing of several gene sequences (Zhang et al., 2016; Gao et al., 2017; Zaman et al., 2019; Li et al., 2021). The type II CRISPR/Cas systems hold an edge over other systems due to rapid continuous advancements with increased precision through numerous Cas protein variants including *dCas9*, *cas12a nickase79*, *fCas980*, *Cpf181* and other comparable nuclease systems While most of the Cas systems rely on the NHEJ DNA repair mechanism, the newly added tools such as base editing (Li G. et al., 2019) and prime editing (Anzalone et al., 2019) provide precise DNA base modification without induction of double strand breaks (DSBs). Although these have been largely exploited in mammals so far, they offer immense opportunities in agriculture and allied fields as well. Another factor influencing the editing efficiency of these tools is the delivery of editing components (Kang et al., 2020). While *Agrobacterium* transformation is the most stable delivery method for the development of edited plants (Sardesai and Subramanyam, 2018), several non-tissue culture-based delivery mechanisms have also been developed that can overcome the limitations of recalcitrancy in crops (Mahas et al., 2019). Moreover, CRISPR/Cas technologies together with rapid generation turnover (RGT)/speed breeding or double haploids are increasingly emerging to be more efficient in developing elite cultivars with safety, precision and speed (Hickey et al., 2019).

In the drylands, particularly in South Asian and Sub-Saharan African countries that have very limited cultivable arable land, little space is left for further crop expansion (Zdruli, 2014). This necessitates the broadening of crop diversity and reducing the burden on certain crops. Several neglected or underutilized crops are grown traditionally in their native environments and are more adapted to marginal farming, that often have high nutritional value with rich genetic diversity (Jacob et al., 2018). The potential of underutilized crop is increasingly being recognized due to their superior trait qualities such as tolerance to biotic and abiotic stress for sustainable agriculture.

## Extending the CRISPR/Cas toolbox for genome editing applications

The CRISPR/Cas protein endonuclease originates from several bacterial species including *Staphylococcus aureus*, *Streptococcus thermophilus*, *Francisella novocida* out of which *Streptococcus pyogenes* is the most widely used source. The SpCRISPR/Cas9 system predominantly recognizes protospacer adjacent motif (PAM) (5′-NGG-3′) and cleaves target DNA just three to four bases upstream of a PAM sequence to create blunt-end DSBs (Cong et al., 2013; Zhang et al., 2021). Several alternative Cas variants such as *dCas9*, *CRISPRi*, *iCas9*, *nickase79*, *fCas980*, *Cpf181*, *C2C2*, *13B*, *Cpf1*, etc. and other comparable nuclease systems have also been developed (Shmakov et al., 2017; Ghorbani et al., 2021). These Cas variants not only offer reduced off-target effects, but also provide higher precision in genome editing applications (Konermann et al., 2018). Among all Cas proteins, the type VI system has relatively simple and exclusive targets for RNA editing (Abudayyeh et al., 2017; Cox et al., 2017; Yan et al., 2018; Burmistrz et al., 2020). Faster customization of Cas variants has increased the target recognition capabilities resulting in multi-fold increase in precision and significantly lowered the off-target effects (Table 1).

**TABLE 1:** Characteristic features of Cas variants and new genome editing tools.

Type of crop	Crop	Region grown	Area of production (m ha)	References
Cereals	Pearl millet (*Pennisetum glaucum*)	South Asia and Africa	34.79	Kumara Charyulu et al. (2014)
	Kodo millet (*Paspalum scrobiculatum*)	South-east and South Asia, Western Africa	0.2	Vetriventhan et al. (2020)
	Finger millet (*Eleusine coracana*)	Africa and South Asia	4–4.5	Vetriventhan et al. (2020)
	Foxtail millet (*Setaria italica*)	North America, Africa, South east and South Asia	0.72	Vetriventhan et al. (2020)
	Proso millet (*Panicum miliaceum*)	North America, Africa, South east and South Asia	1.37	Vetriventhan et al. (2020)
	Teff (Eragrostis tef)	South Africa, Australia, South East Asia and South Asia	NA	Vetriventhan et al. (2020)
	Fonio	Africa, South east Asia	0.96	Vetriventhan et al. (2020)
Legumes	Cowpea (*Vigna unguiculata*)	South Africa, West and central Africa	12.5	Ngalamu et al. (2015)
	Pigeonpea (*Cajanus cajan*)	South Asia, South Africa, West and central Africa, South East Asia	4.6	Saxena et al. (2015)
	Tepary Bean (*Phaseolus acutifolius*)	Sub-Saharan Africa, North America	NA	Jiri et al. (2017)
	Bambara groundnut (*Vigna subterranea* L. Verdc)	Sub-Saharan Africa and South Asia	0.25	Majola et al. (2021)

Table: Major underutilized crops and their area covered under dry regions in the world.

Based on the constitution of effector protein, the CRISPR/Cas system is broadly classified into two major classes that have been further divided into six types (I–VI) and 33 sub-types (Makarova et al., 2020). While in the class I system (types I, III, and IV) the effector consists of multiple proteins, the class II system (II, V, and VI) compromises of a single effector with CRISPR RNAs (crRNAs) (Koonin et al., 2017; Chen et al., 2019). The class II system have been shown to have more flexible applications in inducing the sequence variations such as knock-ins, knockouts, exchange, genetic screening, imaging etc. (Tang and Fu, 2018). Within the class II, the CRISPR/Cas9 system has shown tremendous practical applications over others in a range of plant species from model systems to crop plants for efficient introduction of various traits such as disease resistance (Oliva et al., 2019), nutrition (Ku and Ha, 2020) and climate-resilience (Tripathi et al., 2019).

### Base editing

While the evolution of CRISPR as a tool is remarkable and each shortcoming has been overcome with even more novel editing technologies, achieving precise single base DNA editing is an arduous task. Recently developed editing tools such as base editing (BE) ensure precise single base changes without the involvement of DSBs, HDR and donor DNA templates for selected irreversible nucleotide base substitutions at target sites. Technically, the base editing system mainly comprises of catalytically impaired Cas protein, guide RNA, and a nucleobase deaminase domain.

Continuous advances in base editing tool offers improved editing efficiency or specificity or both by adding the base-edit repair inhibitor, a glycosylase inhibitor, to the fusion protein and modifying the Cas proteins (Marx, 2018). Base editing has several advantages over the existing CRISPR/Cas technologies and has been successfully carried out in several plant species. These include rice, wheat, maize, potato, watermelon, cotton, tomato, and *Arabidopsis* genomes (Chen et al., 2017; Hess et al., 2017; Yang et al., 2017; Zong et al., 2017; Tian et al., 2018; Monsur et al., 2020; Tra et al., 2021) for various traits including nitrogen use efficiency (Lu and Zhu, 2017) and herbicide resistance (Shimatani et al., 2017; Li C. et al., 2018; Monsur et al., 2020). Base editors also offer the disruption of genes by creating early stop codons or inducing transcript mis-splicing in plants (Veillet et al., 2019). While the ongoing base editor endeavors are constantly being improved to adjust to a wide range of crops (Mishra et al., 2020; Sretenovic et al., 2021), using appropriate Cas variants along with CBE and ABE base editors could broaden the horizon for crop improvement besides lowering the off-target effects.

### Prime editing

Several genome editing tools encounter limitations with respect to the precision and utilization of the modified customized sequence simultaneously at the target site and perform single/few base substitutions. To overcome this, “prime editing” (PE) method is a “search-and-replace” system can alter the new genetic information directly at the targeted site without any DSBs or template DNA (Anzalone et al., 2019). Prime editors could efficiently develop all possible base conversions and small indels in a wider targeting range with limited off-target efficiency (Anzalone et al., 2019) and hold great promise for precision crop breeding. The PE components have been optimized to increase their efficiency and deployed in wheat and rice to generate several types of single base substitutions, multiple base substitutions and indels (Lin et al., 2020).

While the prime editing is less efficient than base editing for generating transition point mutations in plants, it generates transversion changes and all single base substitutions that cannot be made with other genome editing tools (Marzec and Hensel, 2020) that are important for applications in a range of crops. So far, most of the success has been achieved in monocots (Li and Xia, 2020a; Hua et al., 2020; Xu et al., 2020; Ren et al., 2021), with tomato as an exception among the dicots where editing of three genes *ALS2*, and *PDS1* was achieved at a frequency of 6.7% and 3.4%, respectively (Lu et al., 2021).

## Epigenomic editing

Since the molecular basis of crop improvements is governed by both genome and epigenome of the plant (Kakoulodou et al., 2021), it is important to integrate them for realizing incremental genetic gains for improved adaptations and sustainable agriculture. CRISPR-based technologies are facilitating accelerated precision breeding, and epigenome editing is the next step in this direction to fast track the breeding process without the risk of genome instability and off-target effects. Since epigenetic modifications such as DNA methylation and histone modifications affect the expression of genes, with emerging knowledge on the functioning of epigenetics in plants, several efforts are ongoing for developing tools and technologies, thereby targeting epigenetic modifications that cause heritable changes. Epigenetic changes like modulation of chromatin, histone, cell differentiation, development and senescence have been shown to be involved in ensuring the survival of plants under stressful environments by enabling the plans to remember past stress events and dealing with these in the future, often referred to as “plant stress memory” (Singh et al., 2021; Sun et al., 2021; Shin et al., 2022). For example, methylation of histone H3 lysine 4 (H3K4) is involved in the persistent expression not only of high temperature-responsive genes, but also as hyper-induction of such genes during repeated heat stress treatments (Lämke et al., 2016). An inducible system for epigenome editing has recently been reported in *Arabidopsis* that uses a heat-inducible dCas9 to target a JUMONJI (JMJ) histone H3 lysine 4 (H3K4) demethylase domain to a locus of interest, the *APX2* gene in this case that showed transcriptional memory after heat stress (Oberkofler and Bäurle, 2022). Such newer tools enable targeted manipulation of epigenetic characters that could be used to specifically modify plant phenotype or to elucidate the relationship between the epigenome and transcriptional control (Hilton et al., 2015; Moradpour and Abdulah, 2020).

Emerging tools such as epigenetic QTLs or epigenetic single nucleotide polymorphisms tools also offer opportunities for activating or repressing candidate gene(s) or pathway(s) for trait improvement in crops, which could lead to the development of a new, efficient, and transgene-free breeding methods (Bilichak and Kovalchuk, 2016). While these new technological advances have shown the possibility of exploitation of epigenetic variation in crop breeding and acceleration and more efficient creation of climate-smart crop varieties, more work is needed in species beyond the model plant systems to gain a more comprehensive understanding of the mechanisms inducing and stabilizing epigenetic variation. In context of the underutilized crop plants, further studies are needed for identifying the specific traits and the association of stress-induced gene expression changes with alterations in DNA methylation and histone modifications, the mode of inheritance of these modifications, and their adaptive value (Chinnusamy and Zhu, 2009). In one such effort (Veley et al., 2021) demonstrated that methylation to the TAL20 effector binding element within the *MeSWEET10a* promoter in cassava using synthetic zinc-finger DNA binding domain prevented TAL20 binding, blocking the transcriptional activation of *MeSWEET10a* displaying increased resistance to cassava bacterial blight (CBB). This offers potential opportunities for editing crop epialleles for adaptation traits. Nonetheless, this will require combined and multidisciplinary efforts in different areas of plant science and better integration of epigenomic data obtained in different crops.

## Delivering genome editing components

The indirect and direct methods of delivery of genome editing reagents have been extensively and successfully used in several crops. Direct delivery methods such as *Agrobacterium* or particle bombardment possess persistent challenges in crops where efficient transformation systems are not available. While these plant transformation methods are cost-effective, convenient, and easily available in laboratories, their delivery efficiencies remain highly dependent on several factors such as type of explant, *Agrobacterium* strain, genotype, construction of independent editing reagents in multiple binary vectors etc. To overcome these drawbacks, direct methods of gene delivery, such as protoplast transfection, virus-mediated, RNP-based, meristem induction, lipofection-and PEG-mediated protoplasts and usage of aiding elements such as special peptides and nanoparticles have been developed and adopted (Ran et al., 2017; Nishizawa-Yokoi and Toki, 2021).

ZFNs, TALENs, and CRISPR have been successfully employed for gene knockout studies using protoplast transfection in different crops by using polyethylene glycol (PEG). However, the primary disadvantage of this technique lies in its inability to transform all plant species, especially monocots. While DNA-free genome editing methods have been obtained by delivering CRISPR/Cas reagents as *in vitro* transcripts or ribonucleoproteins (RNPs), these can give rise to multiple copies of the same gene causing undesirable altered expression (Liang et al., 2017).

More recently, nanoparticles (NPs) have gained pace as delivery vehicles since they can be designed according to the type of tissue and organism of interest (Ahmar et al., 2021). For example, mesoporous silica nanoparticles (MSNs) have been used to deliver the Cre recombinase into maize cells, leading to recombination of *lox* P sites and *DsRed2* expression (Martin-Ortigosa et al., 2014). NPs are highly stable and are flexible in terms of size, shape and distribution as carriers. Like NPs, polymers have been exploited as carriers due to their wide availability. Encapsulation or complexation with polymers, both synthetic and natural, can protect the components from enzymatic degradation and functionally activate them to bind to specific receptors for enhanced targeting. Additionally, lipid molecules have been effectively employed as delivery vehicles. Lipofectamine, a popular, commercial lipid reagent, has been utilized to deliver gene-editing proteins (Zuris et al., 2015).

Cas9 RNPs, containing negatively charged gRNA molecules quickly form a complex with cationic lipids. Nucleic acids have been exploited to function as polymeric substrates for Cas9 RNP delivery. However, further modification in the polymeric coating is vital to ensure that degradation through cellular pathways and enzymes does not occur. An alternative to encapsulation is the modification of the protein and nucleic acid. Cell-penetrating peptides (CPPs) are short peptide sequences that can penetrate the cell membrane easily. They can be conjugated with Cas9 protein and gRNA for enhanced delivery. However, these peptides do not protect the protein from protease degradation within the cell and can be complexed with other delivery methods. Further, nuclear localization sequences (NLSs) which are sequences synthesized in the cytoplasm for tagging proteins and transported into the nucleus are also being explored. They are poly-arginine/lysine and behave as signal molecules attached to proteins for nuclear transport. As Cas9 needs to be transported into the nucleus, NLS are excellent agents for delivery by synthesizing proteins containing NLS or encoding into the Cas9 construct (Glass et al., 2018). While most of these methods are still prevalent, they possess shortcomings, making them inadequate for efficient editing.

Tissue-culture-based techniques require plants to regenerate from transformed cells/explants which makes the procedure highly time consuming. Also, transformation protocols are genotype-dependent and effective protocols are not established for recalcitrant crop species. Therefore, new techniques have emerged that eliminate the need for traditional tissue culture techniques and *in planta* methods like floral dipping (Ji et al., 2020) and anther culture (Han et al., 2021). The gene-edited somatic cells are re-programmed into meristematic cells by expressions of developmental regulator (DR) genes, such as *WUSCHEL2(Wus2)* and *BABY BOOM* by using the genome-editing machinery. It has been demonstrated that the ectopic expression of DRs like *Wus2*, *SHOOT MERISTEMLESS(STM)* or *MONOPTEROS (MP)* induces the development of meristem-like structures in *Arabidopsis.* Additionally, the co-expression of Wus/STM and CRISPR/Cas9 cassette in *Nicotiana benthamiana* to target *phytoene desaturase* (*PDS*) gene was also carried out. The meristems later develop into shoots (Maher et al., 2020) demonstrating comparable mutation frequencies.

In another study, abundant shoots were successfully obtained through *in planta* transformation protocol in tobacco, where CRISPR/Cas9 expressing plants growing in soil were injected with *Agrobacterium* cultures carrying appropriate DR and sgRNA in the sites where meristems were removed. This study demonstrated altered development of edited somatic cells, induction of meristems and their growth in fertile plants through co-expression of DRs and CRISPR/Cas9 system. Similar efforts have been made with editing the commercial varieties of wheat (Liu et al., 2021) that completely circumvented the need for tissue culture procedures to obtain genome-edited plants. The exclusion of tissue culture-based genome editing reduces cost, labor and amplifies efficiency. *De novo* meristem induction-mediated genome editing is still quite novel in terms of research. Additionally, abnormal growth has also been observed due to the constitutive expression of DRs. This can be overcome by inducible expression of the DRs. While the *de novo* shoot meristem induction has been exploited in grape, potato, and tomato crops, their feasibility in crops with heritable mutations is yet to be explored. This utility of the technique also needs to be extrapolated to staple food crops (Ji et al., 2020; Chennakesavulu et al., 2021).

Tissue culture-free genome editing technique has also been studied in meristem tissues developed from imbibed embryos of wheat seed (Hamada et al., 2018). The infection of meristematic tissues by virus (TRV) expressing SpCas9 protein was reported in tobacco wherein, the gRNA was fused with *Arabidopsis Flowering Locus T (FT)* mRNA, resulting in mutation of up to 65%–100% in the edited plants. Further, TRV was not detected in the progeny, subsequently protecting the progenies from any viral effects. This *in planta* gene-editing technique showed tremendous promise as it successfully generated small mutations in the gene. However, the method exhibited certain shortcomings in terms of identifying species-specific effective viral vectors and the gRNA-FT translocation abilities, highlighting the need for identification and characterization of viruses that infect meristematic tissues (Ellison et al., 2020; Kim et al., 2020).

## Genome editing for underutilized dryland crops: Progress and prospects

The drylands make up to 41% of the total global land area and are characterized by low precipitation and drought. Comprising of mainly parts of western, central and southern United States, East and west Africa, the middle east, parts of Indian subcontinent and the central deserts of Australia, these areas are prone to environmental factors such as wind erosion, mineral weathering, and low fertility (Hyman et al., 2016). The climate change has made imminent the need for adapting climate-smart crops as well as growing more resilient underutilized having low water requirements. A comprehensive overview of the major crops grown in drylands and their area of production is given in Table 1.

To mitigate the challenges of agricultural productivity in the underdeveloped and developing countries and to make agriculture sustainable under diverse climatic conditions, it is critical to develop transformative strategies for breeding pipelines by using the new breeding acceleration techniques. While tweaking selection accuracy and intensity can lead to minor improvements in a breeding context, generating novel and useful genetic variations and rapidly fixing the traits facilitate crop genetic gains can allow faster turnover of improved cultivars for accelerated delivery of improved varieties to farmers. The availability of reference genomes and ever-increasing re-sequencing data has significantly advanced breeding applications and allowed to capture the genomic diversity and its effective mining. This revelation has helped in understanding genes and the mechanisms underlying various biotic and abiotic stress responsiveness, quality, besides nutrition and plant architecture parameters, thereby aiding considerably in developing crop species with adaptive and resilient traits.

**FIGURE 1:**
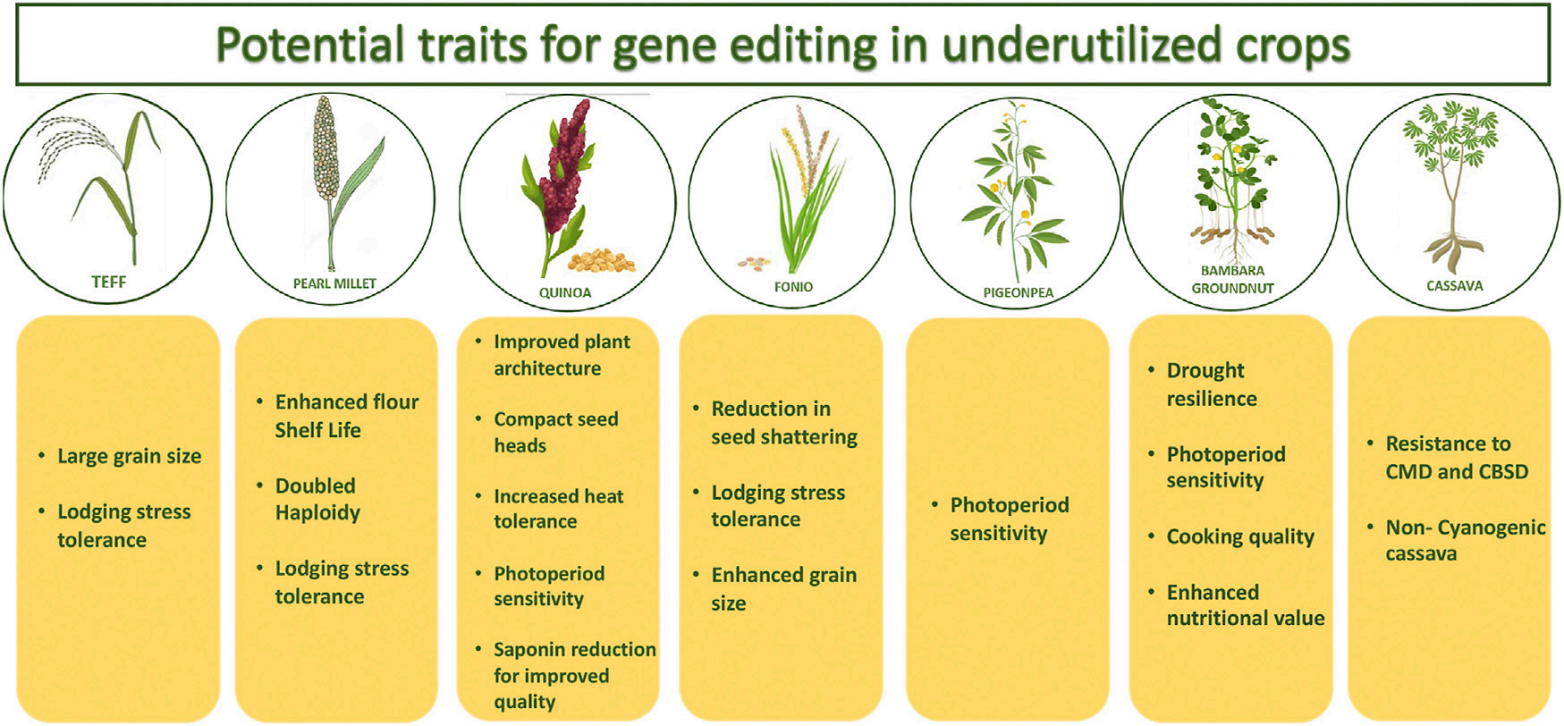
Schematic representation of the potential traits which can be explored in underutilized crops using gene editing technology.

Genetic resource collections that are deemed to harbor a wealth of undisclosed allelic variants are being unlocked by identifying allelic variation of relevant traits within these collections. The enormous genetic diversity present in wild species or landraces of crops as a source of allele-mining could very well be utilized and translated to elite backgrounds using genome editing tools, thereby potentially expanding the crop germplasm pool. Optimization of these tools in the underutilized food crops like sorghum, millets, groundnut, beans, cowpea, teff, banana, cassava etc. that are primarily cultivated by the poor and resource-poor farmers of the drylands would lead to huge impact in achieving the global food and nutritional security goals. This will not only accelerate the pace of the ongoing research but will potentially enable a disruptive reduction in cost for development of both farmer- and consumer-centric traits/products in these important crops. A comprehensive overview of the potential traits which can be explored in underutilized crops using genome editing has been given in Figure 1. Some of the important subsistence crops and traits of the drylands that could potentially be addressed through genome editing methods are discussed below.

For success with precision breeding, successful genetic transformation of underutilized crops is one of the prerequisites for delivery of recombinant DNAs as well as genome editing components into the plant cells that regenerate into whole plants. While *Agrobacterium* transformation has been successfully developed for almost all staples, there has not been a great deal of progress in improving the transformation frequencies for a many underutilized crops. Currently, transformation competent methods have been developed for crops such as finger millet (Ceasar and Ignacimuthu, 2011; Hema et al., 2014); foxtail millet (Ceasar et al., 2017; Santos et al., 2020) and pigeonpea (Sharma et al., 2006; Ghosh et al., 2017). While genetic transformation of several underutilized legumes is still in its infancy, stable and reproducible transformation system based on callus derived from floral buds and cotyledonary node region is available for tepary bean (Dillen et al., 1997; De Clercq et al., 2002; Zambre et al., 2005). In bambara groundnut, an efficient system for *in vitro* shoot induction from cotyledons derived from mature seeds has been established to subsequently exploit transformation technologies in this important legume (Koné et al., 2011). In cassava, transformation systems have been developed and much progress has been made in the development of *Agrobacterium*-based transformation protocols (reviewed by Liu et al., 2011). Similarly, in quinoa, a rapid transformation system was established using hairy roots obtained from cotyledon-nod with hypocotyl, cotyledons and hypocotyl pieces at a transformation efficiency of 32%–68% (Wang et al., 2021). Concerning pearl millet, while several reports showed transient expression of the reporter genes in transformed calli (Ramineni et al., 2014), barring a report of Ignacimuthu and Kannan (2013), not many stable transformation methods have been reported. Owing to the inefficiencies and inconsistencies in the published protocols for several crops, several non-tissue culture-based approaches are being optimized for transformation that do not depend on the regeneration of adventitious shoot buds (Martins et al., 2015).

### Green revolution traits-millets

The green revolution (GR) evolved from specific requirements in nutrition and yield productivity primarily enabled by vast genetic resources of the gene banks. The transfer or replacement of dwarfing genes into cultivated crops such as rice and wheat resulted in shorter straws allowing diversion of more nutrients into grain, besides making heavier ears that allowed higher yields and better agronomic performance (Gale and Youssefian, 1985). The GR traits can be exploited for teff (*Eragrostis tef*) and finger millets that are known to flourish and grow well in East African climatic and soil conditions (Tadele and Assefa, 2012), where lodging leads to a considerable loss in their harvest. In rice, several quantitative trait loci (QTLs) that contribute to lodging stress tolerance have been identified and successfully integrated into the development of improved varieties (Liu et al., 2018). Similarly, the revolutionary gene, *sd-1*, that encodes for *gibberellin-20 oxidase*, provided rice varieties with lodging resistance without affecting the grain quality (Monna et al., 2002; Sasaki et al., 2002; Spielmeyer et al., 2002). Similarly, in wheat *Rht-B1* (*reduced height-B1*) and *Rht-D1* genes imparted lodging tolerance via dwarf plant development (Würschum et al., 2017). In maize, a close homolog of *dw3*, *Br2*, was identified which is an ATP-binding cassette-type B1 (ABCB1) auxin efflux transporter (Hilley et al., 2017).

The dwarfing trait in sorghum has been bred using *dw* (*1–4*) genes (Multani et al., 2003). The possibility of CRISPR/Cas9-mediated targeted gene modification has been demonstrated to be efficient in sorghum (Jiang et al., 2013b; Char et al., 2020). Editing of an alpha-Kafirin gene family that form protein bodies with poor digestibility was shown to increase digestibility and protein quality in sorghum grains following the CRISPR/Cas9 approach (Li A. et al., 2018) The diploid genome of foxtail millet (*Setaria italica*) has recently been sequenced and annotated (Bennetzen et al., 2012; Jia et al., 2013) that could serve as a model system for C4 plants. More recently, Cheng et al. (2021) have reported the induction of haploid embryos through seed by CRISPR-Cas9 mediated mutagenesis of the *SiMTL* gene that is orthologous to the maize MATRILINEAL/NOT-LIKE-DAD/PHOSPHOLIPASE A (*MTL/NLDZmPLA*) gene that generated haploids in maize (Liu et al., 2017). This study paves ways for utilization of the double haploid breeding for enhancing genetic gains in dryland cereal crops.

The identification of height related and root architecture genes in the dryland cereal crops provide a foundation for evolutionary and functional analysis of specific proteins defining a comprehensive view of *Rht*, *dw3* or *Br2* family genes (Zhu et al., 2012; Zanke et al., 2014; Wei et al., 2018) in nutri-cereals such as millets and teff. For example, double knockout maize mutants of *ZmPHYC1* and *ZmPHYC2* created using the CRISPR/Cas9 technology displayed a moderate early-flowering phenotype under long-day conditions, while the overexpression of *ZmPHYC2* exhibit a moderately reduced plant height and ear height (Li et al., 2020b). A recent review summarizes genome editing efforts on plant architectural phenotypes in cereals and their manipulation to optimize their architecture towards the concept of ideotype for crop improvement (Huang et al., 2021).

### Cassava-disease resistance and quality traits

Cassava (*Manihot esculenta*) is a very important crop which is not only vital to food security in tropics and subtropics, but also a predominant raw material of starch industry (Zhou et al., 2015). Cassava, an important staple food, is grown globally for the calories, of which it provides up to 50% intake of calories (Bredeson et al., 2016) for over 800 million people worldwide (Prochnik et al., 2012). Grown in marginal environments and provides for one of the most important sources of carbohydrate globally, this gluten-free carbohydrate source has seen up to 60% increase in global harvest between 2000 and 2012. There is a continuing need to improve the yields and adaptation of elite cassava varieties (Bull et al., 2018).

Cassava encounters some of the most devastating diseases caused by brown streak virus and cassava mosaic virus causing up to 50% crop yield losses (López and Bernal, 2012). Cassava mosaic virus disease (CMD) is caused by three innate types of Gemini virus; *CMD1*, known to be recessive and governed by multiple genes (Rabbi et al., 2014), *CMD2* possesses a single dominant locus on chromosome number 12 (Akano et al., 2002; Rabbi et al., 2014; Wolfe et al., 2016) and *CMD3* contains a QTL conferring resistance (Houngue et al., 2019). The development of resistant cultivars using somatic embryogenesis in CMD1 was ineffective due to loss of resistance in subsequent generations (Beyene et al., 2016). Hence, *CMD2* and *CMD3* could be the potential candidates for further exploitation via CRISPR/Cas-mediated site-specific targeting (Baltes et al., 2015; Bart and Taylor, 2017). Similarly, simultaneous CRISPR/Cas9-mediated editing of two isoforms of host translation factors, nCBP-1 and nCBP-2 conferred significant resistance to Cassava brown streak disease (CBSD) (Gomez et al., 2019). Suppression of interaction of viral genome-linked protein (VPg) with mutant alleles ncbp-1, ncbp-2, and ncbp-1/ncbp-2 resulted in delayed and attenuated CBSD aerial symptoms, as well as reduced severity and incidence of storage root necrosis.

In addition to disease resistance traits, herbicide tolerance was achieved in cassava by deploying HR and NHEJ DNA repair pathways (Hummel et al., 2018). For quality traits, efforts have been made to improve the quality of its starch, for developing suitable starch properties for cooking and processing. CRISPR-Cas9 mediated targeted mutagenesis of two genes protein targeting to starch (*PTST1*) and granule bound starch synthase (*GBSS*, involved in amylose biosynthesis), have been reduce amylose content in cassava root starch (Bull et al., 2018). In addition to improving the quality of starch, several research groups have been making efforts to develop a cynogenic-free cassava by using gene editing approaches for blocking the production of cyanide. Cassava contains potentially toxic levels of cyanogenic glycosides (Linamarin and Lotaustralin) which if not efficiently removed through processing, may cause various neurological disorders and in some cases may be fatal. The biosynthetic pathway of cyanide in cassava was already well understood and *CYP79D1/D2* gene that encode two cytochrome P450s catalyze the first-dedicated step in cyanogenic glycoside synthesis. Selective inhibition of this gene by antisense expression in leaves and roots have demonstrated a 99% reduction in root cyanogen levels providing road map for using genome editing methods for complete knockdown (Otun et al., 2022).

### Grain size and plant architecture traits in teff and fonio

Another set of dryland crops including teff (*Eragrostis tef*) and fonio (*Digitaria* sp.) which despite their applications in food and feed, high nutrient content and high durability, are among the most under-utilized crop species in the African region (Lee, 2018). Teff is considered as “risk crop” due to its high adaptivity even under extreme conditions of drought and waterlogging and is now in high demand as a forage crop (Miller, 2007). Fonio, on the other hand is considered as the “*grain of life*” and is known for its high nutrient content and contains all 20 amino acids including methionine and cysteine (NRC, 1996; Taylor, 2017).

Mining the homologs of rice genes associated with grain size and weight (Li et al., 2011) in teff could be an effective way of achieving larger grain size in this nutritious cereal and will be a crucial step towards their genetic improvement (Valentine et al., 2017). Fonio shares a close synteny with sorghum, and mutations in genes such as DeSh1-9A, that have shown partial selective sweep but reduced seed shattering in sorghum, can also result in another beneficial architectural trait (Abrouk et al., 2020).

Improving the plant architecture of these underutilized crops is a major breeding goal towards the concept of “ideotype for crop improvement” (Huang et al., 2021). Green revolution saw transfer or replacement of dwarfing genes into cultivated crops such as rice and wheat resulted in shorter straws allowing diversion of more nutrients into grain, besides making heavier ears that allowed higher yields and better agronomic performance (Gale and Youssefian, 1985). There is a vast potential to exploit GR traits for dryland cereals such as teff and finger millets, the major staples of east Africa (Tadele and Assefa, 2012), where lodging leads to a considerable loss in their harvest. Several quantitative trait loci (QTLs) that contribute to lodging stress tolerance have been identified and successfully integrated into breeding programs for improved rice and wheat varieties (Monna et al., 2002; Sasaki et al., 2002; Spielmeyer et al., 2002; Würschum et al., 2017; Liu et al., 2018). Genetic variations in *sd1* and *RHT* genes (Peng et al., 1999) have shown significant lodging tolerance in these major staples and translating these to crops like teff and fonio offer tremendous opportunities. More recently, Beyene et al. (2022) created CRISPR-induced knockout mutations in the tef orthologue of the rice *SEMIDWARF-1* (*SD-1*) gene that conferred semi-dwarfism and significantly higher resistance to lodging resistance in tef. Similarly, homologs of *OsSPL14* (squamosa promoter binding protein-like 14) gene and microRNA “*OsmiR397*” that have been reported to confer panicle branching trait in rice has potential to be explored and targeted in teff and fonio (Miura et al., 2010; Zhang et al., 2013; Numan et al., 2021).

In addition, the grain size of teff and fonio has been a major cause of reduced yield where not much progress has been made in terms of hybrid development through conventional breeding. However, since this trait is being extensively explored in other staple cereals such as maize and rice and with the availability of annotated genomic sequences of sorghum and foxtail millet there are emerging opportunities to identify candidate genes that might share genomic synteny with teff and fonio (Saha et al., 2016; Ayenan et al., 2018). Functional analysis and identification of homologs of these genes in teff and fonio will further help to form a basis for developing lines with enhanced grain size.

### Quality traits-quinoa and pearl millet

Quinoa (*Chenopodium quinoa*), a pseudo cereal belonging to *Amaranthus* family originated in the Andean region grows in the marginal lands is one of the best food choices due to its balanced amino acid profile, vitamins, minerals, ions, and antioxidants, quinoa received a “superfood” status and contributes to the economic and global nutritional security (Vega-Gálvez et al., 2010). However, despite being nutri-climate-resilient, it is still an underutilized crop with major breeding objectives including, improved plant architecture, compact seed heads, increased heat tolerance, photoperiod and heat sensitivity. A well-annotated and high-quality reference genome sequence has recently been made available (Jarvis et al., 2017), thereby offering opportunities for allele mining for trait prospecting efforts. However, precision breeding in this crop requires establishing genome-scale engineering platforms and toolkits to understand gene functions and their interactions.

Quinoa seeds contain a mixture of triterpene glycosides called saponins that contribute to plant growth to a certain extent. However, this anti-nutritional property must be removed prior to human consumption as these saponins cause hemolysis in humans and a bitter flavor that are undesirable traits. Reducing or eliminating the saponin through physical and traditional approaches is costly and often water-intensive and negatively affects the quality of nutritional elements. Identification of the candidate genes and their genetic variations underlying the saponin biosynthetic pathway have been investigated in different germplasms of quinoa with the help of existing sequencing data (Jarvis et al., 2017) that would provide a platform for further studies in the generation of genotypes with sweetness and low saponin and their introgression into commercial varieties.

While CRISPR/Cas tool provides a robust platform for targeted quinoa breeding, the lack of an efficient transformation system in quinoa would be another objective in developing the next generation quinoa plants. Genetic transformation methods, including *Agrobacterium*-mediated transformation, hairy root and leaf agroinfiltration techniques have been used for quinoa (Wang et al., 2021). However, the transformation efficiency at this stage may not be sufficient for any meaningful genetic engineering and genome editing strategies. Nevertheless, the possibility of generation of transformed quinoa plants through *Agrobacterium*-mediated transformation is not a vague reality. This technique can be improved further by using the booster genes such as *WUSCHEL*, *BABY BOOM* and *LEAFY COTYLEDON1* which have been previously known to improve the transformation efficiency in other crops like maize and sorghum (Nelson-Vasilchik et al., 2022). The *de novo* induction of meristems could also be an alternative approach along with the expression of booster genes to avoid complications in tissue-culture strategies. Other challenge in quinoa genome editing could be due to its allotetraploid nature, where targeting all four copies of these genes could be challenging. Multiplex genome editing would be an ideal solution in this scenario as it has been carried out in other polyploidy crops such as wheat, canola, sugarcane, and banana (Vats et al., 2019).

In addition to quinoa, another food and nutritional security crop, pearl millet (*Pennisetum glaucum*) that grows in some of the most hostile-to-farm landscapes despite its many superior attributes, has an unsolved quality issue of flour rancidity, posing a hindrance to its wider acceptability. Rapid development of off-flavor in pearl millet flour within 5–7 days of milling hinders the commercial use of this crop besides creating additional drudgery for women of the household, as the amount that can be pounded is limited to a few days of household use, thereby necessitating that the grain be milled immediately prior to use. A collaborative effort between the CGIAR and industry outlined a direct mechanism for hydrolytic and oxidative rancidity in millet flour, allelic variation two candidate lipase genes, Pg*TAGLip1* and Pg*TAGLip2* were identified, that correlated with the rancidity profile, confirming their function. Mutations in these key TAG lipases in pearl millet have potential in protection of lipids from TAG hydrolysis and fatty acid oxidation, leading to a reduction in off-flavour volatiles (Aher et al., 2022). In addition, since pearl millet has abundance of unsaturated fatty acids (>78%) representing the reactive center that produces odor-active volatiles, major markers for lipid oxidation (Sharma, 2015). Hence, shifting the fatty acid profile in pearl millet from poly unsaturated fatty acids (PUFA) to monounsaturated ones (MUFA) by generating inactive or partially active *Fad2* alleles, will serve to not only increase the shelf life but also deliver health dividends because of the positive health benefits of the monounsaturated fatty acids.

### Abiotic stress component traits-Bambara groundnut (*Vigna subterranea*)

Bambara groundnut is an underutilized legume found mainly in the African sub-continent. Due to its high content of complex carbohydrates, unsaturated fatty-acids, minerals such as magnesium, iron, zinc and potassium, fiber, and plant-based proteins, it holds the potential for providing food security through a sustainable approach, especially in the arid and semi-arid region (Olanrewaju et al., 2022). However due to lack of knowledge, appropriate policies and resource limitation, Bambara groundnut is often overlooked and therefore, is categorized as an underutilized crop (Travella et al., 2019).

The first genome sequence of the bambara groundnut was released by Chang et al. (2018) which opened avenues for improvement of the crop through genetic approaches. Major traits of importance in Bambara groundnut are drought-resilience, photoperiod response, cooking quality and time, and nutritional value (Muhammad et al., 2020). Along with this, pipelines of other crops have also been utilized to develop translational frameworks are being used to provide gene orthologues in this legume crop (Popoola et al., 2019). For example, massively parallel signature sequencing (MPSS) strategy employed for expression profile analysis of Bambara groundnut under water-deficit conditions led to the revelation that major transcription factors like MYC, WRKY protein and DREB were absent in the dataset. A recent study assessed the genetic diversity and structure among Bambara groundnut landraces collected across South Africa and other regions in southern Africa using SSR markers for the cultivation and improvement of Bambara groundnut (Minnaar-Ontong et al., 2021). More recently, KUP genes have gained attention for their role in abiotic stress tolerance and hence offer opportunities for precision genetic interventions in Bambara groundnuts. This provides scope for further improvements and genome editing tool has potential to deploy these novel traits and aid precision breeding of Bambara groundnut (Paliwal et al., 2021).

### Photoperiod sensitivity-pigeonpea (*Cajanus cajan* L.)

Pigeonpea is an important climate resilient annual legume grown in parts of Asia, Africa and Latin America grown with other legumes and cereals. Genetic studies on the essential traits of pigeonpea such as maturity, photosensitivity, breeding behavior and disease and pest resistance have implied that the major agronomic traits are mainly additive in nature. The first pigeonpea hybrid was developed in the 1990s based on cytoplasmic-male sterility-based breeding system. Advances in next-generation sequencing (NGS) has revolutionized GAB by facilitating development of markers for unique agronomic traits (Pazhamala et al., 2015; Singh et al., 2020) and have played a significant role in building breeding programs. However, modern technologies such as CRISPR/Cas9 based editing are integral for unravelling mechanisms of other important traits and enhancing pigeonpea program.

Being a short-day legume differential genotypic sensitivity to photoperiod has major implications in adaptation of pigeonpea with respect to latitude, altitude and season. Most of the traditionally grown pigeonpea cultivars and landraces are represented by varieties from the medium- and long-duration maturity groups that mature in 150–280 days. To expand pigeonpea cultivation into new crop improvement programs, the manipulation of flowering time is likely to contribute greatly to crop yields through tailoring of cultivars to specific climates or to changes in climate that are anticipated to occur. Certain SSRs and SNPs have been identified which shed light on the pleiotropic relationship between photosensitivity and flowering time (Bohra et al., 2020).

The manipulation of flowering time is likely to contribute greatly to crop yields through tailoring of cultivars to specific climates or to changes in climate that are anticipated to occur.

However, to accomplish this, an understanding of the genes associated with transition from photoperiodic sensitivity to photoperiodic insensitivity is required. Such knowledge can be used to develop pigeonpea germplasm that can be grown for yield gains under both long- and short-day conditions and provide sustainable production of grain legumes. A recent report provided detailed characterization of the genes involved in photoperiodic regulation of flowering in *C. cajan* offering clues to the role of PEBP (FT) family genes, based on genome-wide analyses and expression profiling. *CcFT6* and *CcFT8*, were identified as probable *Flowering locus T* genes that are responsible for the production of florigen in pigeonpea. While *CcFT6* upregulates under SD in photoperiod sensitive, MAL3 genotype, *CcFT6* and *CcFT8* upregulate in photoperiod insensitive genotype (ICP20338) under SD and LD conditions, respectively. The presence of *CcFT8* as an additional florigen producing gene, having ability to flower in a photoperiod independent manner under LD conditions provide some clues on its photoperiod insensitive nature (Tribhuvan et al., 2020). More recently, two candidate genes coding for pentatricopeptide repeat (PPR) and cell division protein *FtsZ* homolog have been investigated in pigeonpea. These two candidate genes and previously reported genes such as *CcTFL1* and *EARLY FLOWERING3* (Saxena et al., 2017; Varshney et al., 2017) could be validated at a functional level for their specific roles. Tailoring of CcFT8 and other candidate FT genes, using genome editing has a potential to provide answers to the understanding molecular mechanisms associated with the trait. Moreover, precision targeting of the identified candidate genes involved in flowering, would play a crucial role in extending the cropping area of pigeonpea*,* a photoperiod sensitive major grain legume into new cropping systems.

## Accelerating genetic gains through genome editing

*Ex-situ* collection of plant germplasm and its maintenance is crucial to protect the vast genetic diversity in crops that are fast deteriorating due to the development of domesticated cultivars over traditional landraces (Pérez-Jaramillo et al., 2016). Systematic phenotypic evaluation of the available resources would help researchers gain perspective about the underlying potential of these landraces. Genetic gains (Falconer and Mackay, 1996) in a species occurs when the frequency of desirable genes is increased usually achieved by selection of elite parental varieties based upon their phenotypic or genotypic characteristics. Since developing homozygous lines could take at least 10 years through conventional breeding, it alone will not be sufficient to bridge the gap between current level of crop production. Hence the rate of genetic gain has remained considerably low with time (Bhattacharya et al., 2021). Several strategies to create and unlock favourable genetic variations through molecular and genomic approaches including mutation, gene mapping and discovery, transgenics, and genome editing to enhance genetic gains in crops have been reviewed (Xu et al., 2017).

On the breeder’s equation, the rate of genetic gain in any crop is related to the selection intensity that is applied, which is in turn related to the size of the breeding program, the accuracy of the data or the selections made. Essentially it is about the parental selections, genetic diversity, and the breeding cycle time. So, when it comes to breeding cycle time, many breeding programs have a cycle of more than 10 years and some could be even more up to 25 years. So, driving down, breeding cycle time on the denominator has really a massive impact on the genetic gains. That’s when advanced breeding technologies that can improve the accuracy have the potential to create their greatest impact. Since the collective impact is greater than the sum of the parts, a synergistic integration of conventional breeding and gene editing approaches can deliver the highest possible rate of genetic gains bringing down the age of varieties in farmer’s fields.

Additionally, changes in the zygote and germline cells would give rise to further heritable changes which are maintained across generations (Gonen et al., 2017). However, it was observed that to achieve persistent variance, one to two generations of editing were required due to the segregation of non-desirable alleles within the non-edited parents. Genome editing holds the potential for facilitating the identification of essential genetic variations and their deployment in breeding programs. Due to the availability of high-throughput screening technologies, the desirable phenotypes can now be identified and employed in pre-breeding strategies to obtain genetic variations. Such data allows the identification of core traits and sometimes in the discovery of specific genes that could aid in understanding relevant, novel and useful variations in elite varieties (Mascher et al., 2019). An interesting concept which has been proposed is the “re-domestication” of crops using CRISPR/Cas9 mediated knockouts. Such targeted gene modifications are being considered to induce genomic selection as well as transfer beneficial traits between domesticated crops and their wild varieties which otherwise is a time and labor-intensive process (Lemmon et al., 2018).

Additionally, quick domestication of annual crops is a real challenge because the crop would be sown each year in the same agricultural land, thereby deteriorating soil fertility that would eventually lead to lower nutrient and mineral uptake. Therefore, the domestication of perennial crops such as wheat could be a significant steppingstone towards achieving sustainable agricultural practices. Some unsuccessful attempts have been made to turn a wheat variety into a perennial crop by hybridizing with the wild varieties of some grasses. In such cases, the process can be accelerated by CRISPR/Cas tools by targeting the domestication of homologues genes for their successful knockout (Venske et al., 2019).

There is a need to explore the wider domestication opportunities for less researched and invested crops such as sweet potato, groundnut, cassava, teff, fonio, banana and quinoa, which are locally crucial for their extensive nutritional values. However, some undesirable characters such as lower grain yields, sprawling growth and fruit drop limit calls for a more comprehensive cultivation. Therefore, the demonstration to control plant architecture, flower production and grain size by CRISPR/Cas technology in ground cherry, which is semi-domesticated orphan crop opens a wide array of applications of accelerating the genetic gains by editing multiple sites and modifying gene regulation (Lemmon et al., 2018; Zhang et al., 2020).

## Current global regulations for genome edited crops

**FIGURE 2:**
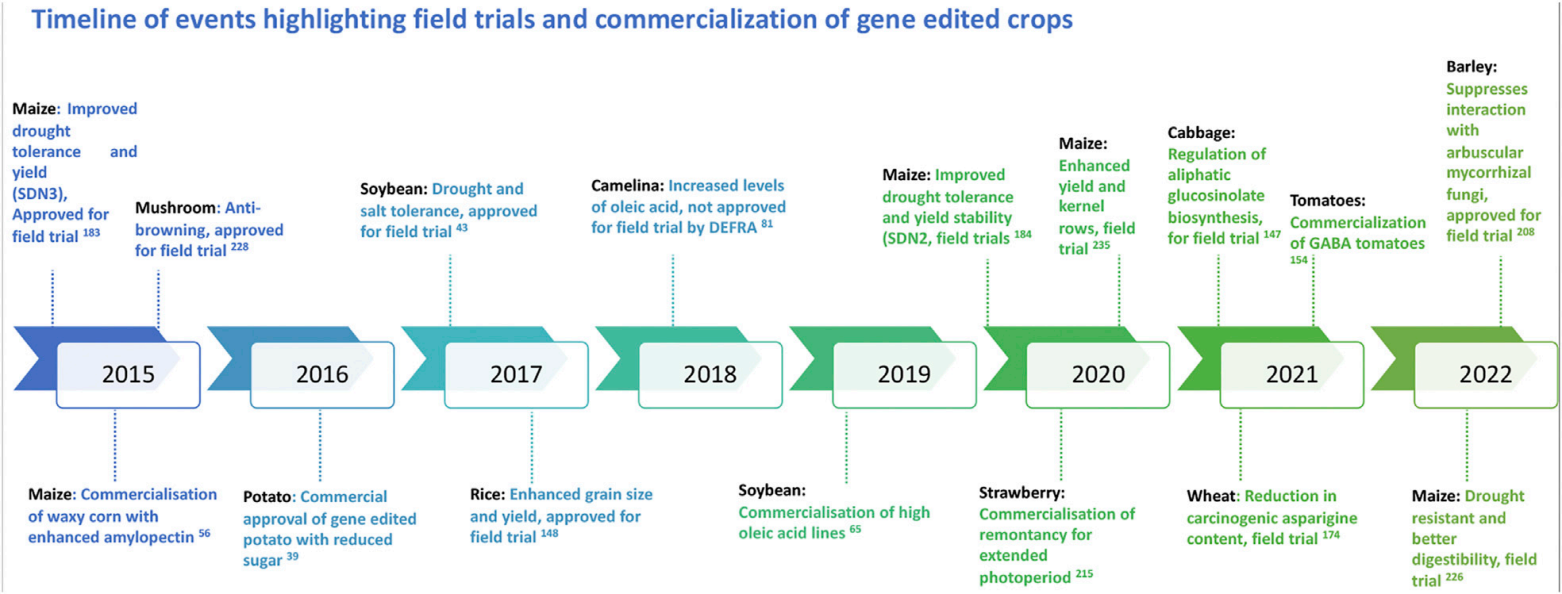
Diagrammatic representation of the timeline of events highlighting the field trials and commercialization of gene edited crops globally.

**FIGURE 3:**
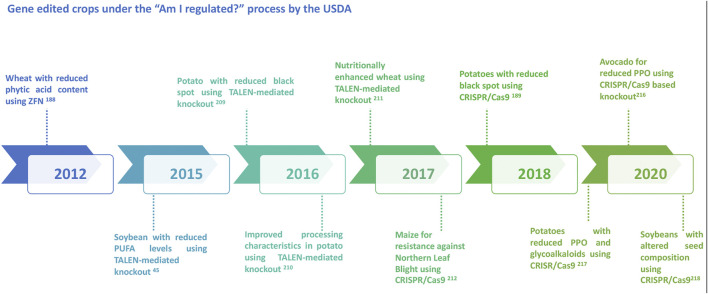
Diagrammatic representation of some of the major gene edited crops approved under the “Am I regulated?” process by the USDA.

With the discovery of genome editing tools, a wide array of applications has been introduced and experimented in various organisms including viruses, bacteria, humans, animals and plants. In the light of recent developments in genome editing, product trials are ongoing in several crops across many countries and regions. While the application of genome editing for genetic gains and crop improvement has a highly potential, it is subject to immense societal resistance. As with any new technology, there are apprehensions around gene editing technologies. To fully comprehend the ethical debates and concerns on genome editing, it is important to understand the process and possible outcomes (Brokowski, 2018; Lassoued et al., 2021). Efficient science communication around the edited traits also may help in improved application and acceptability of these new breeding techniques. Regulatory policies of genome-edited plants in various countries adopt two major frameworks such as the process or the final product. Currently, while very few countries have developed the regulatory frameworks, a majority are yet to develop or declare their regulation process. Decision to either regulate or not to regulate the genome edited crops mainly depend on the type of regulatory system that already exists in a country. A recent review provides an update on the regulatory status of new breeding techniques and biosafety approaches in select countries (Obukosia et al., 2020). Nevertheless, with the evolving regulatory framework on genome editing, certain crops have surpassed the regulations to be now under field trial or on the road to commercialization (Figure 2). The “Am I Regulated” process of the USDA (now SECURE Rule’s Exemption and Confirmation Process beginning on 17 August 2020) allows for developers to determine whether their genetically modified or gene edited organism meets the regulations or not. With the introduction of this process, several inquiries have been submitted to the USDA for gene edited crops of specific traits, some of which have been duly approved (Figure 3).

The United States Department of Agriculture (USDA) declares that genome editing is almost equivalent to conventional breeding and therefore, does not require any regulatory process within the United States. Some speculations from USDA define that gene-edited plants can be considered as a separate category (Menz et al., 2020). In a recent development in the United States , ‘USDA APHIS’ announced the first comprehensive revision to 7 CFR part 340 which is referred to as “SECURE” rule to regulate biotechnology (USDA, 2020a). It provides three very important exemptions for single genetic modifications including products that would be categorized as SDN-1 or SDN-2 in terms of the outcomes of genome editing. The third exemption would also include the introduction of a gene that is known to occur in a plants’ gene pool or allele replacement. In Canada, the regulatory framework is based on the risk of the products comprising a policy of regulating the novelty of new traits in plants or the novel characteristics of new foods or livestock feeds. Hence, whether genome edited products will be regulated or not will depend upon the characteristics of the final product and not on the technology that was used (Smyth, 2017). Till date, two products including the non-browning apples and non-dark spot potatoes developed through gene editing have cleared the regulatory process in Canada.

Argentina’s regulatory process is in accordance with the Cartagena Protocol on Biosafety and evaluated on case-by-case assessment, irrespective of the method used for product development (Lema, 2019). If the edited product is transgene free, the product is classified as non-transgenic and does not require any regulatory process. Countries like Chile, Brazil and Colombia follow the Argentinian model for their regulatory policies, evaluating such products on a case-by-case basis and exempting them from regulation when there is no insertion of a foreign gene (Tsuda et al., 2019).

While in Australia and New Zealand, products developed through CRISPR/Cas9 and other editing tools are excluded from the regulatory process (FSAN, 2018), the European Union (EU) countries follow unique regulatory process where the Court of Justice of the European Union (ECJ) has declared gene-edited crops as subject for stringent regulations as conventional genetically modified (GM) organisms (Laaninen, 2019). Amongst the Asian countries, Japan recently declared that foods derived from genome editing technologies which do not contain any foreign genes and/or fragments are not considered as GMOs and do not require any regulatory clearances (USDA, 2020b).

In India, a recent notification has exempted Site Directed Nuclease (SDN) 1 and 2 types (SDN1 and SDN2) of products which do not carry any vector DNA and are like the products of spontaneous or induced mutations from the transgenic regulation and risk assessment under Rules 1989. Guidelines for the safety assessment of genome edited plants 2022 have been released in May 2022, that define various categories of genome edited plants and determine regulatory requirement for appropriate category and provide the regulatory framework and scientific guidance on data requirement (DBT, 2022).

Similarly, Philippines has moved ahead with, a policy discussion paper under review and consideration on how products of new plant breeding technologies should be treated under existing regulatory regime, the benefits that may be derived and the capacity of the country to utilize such techniques. The policy framework will rely on a case-to-case and crop-to-crop based decision or regulatory pathways which will be the entry point of any genome edited plant products with or without involving the insertion of genes from non-sexually compatible species. However, in regions where the technology and infrastructure has not advanced enough, containment and monitoring measures are expected to be comparatively strict.

In South Africa, SDN-1 involving “small, targeted and untargeted inserts or deletions based on non-homologous end joining (NHEJ)” resulting from ZFNs, MNs, TALENs and CRISPR/Cas and considered to be exempt from GM Act (ASSAf, 2016). The regulatory guidelines for specific countries in Africa are at various stages of development (Travella et al., 2019; Obukosia et al., 2020).

While CRISPR/Cas9 is an inexpensive and flexible technology, international harmonization of the regulatory frameworks needs to be developed to ensure that these are based on sound science and the community of practices developed around the world (El-Mounadi et al., 2020). More deliberative and worldwide conversation is expected to reexamine and rethink about the existing prohibitive rules and devise strategies to grow more logical and specialized overall models for genome editing applications in food and agriculture for betterment of farmers’ livelihoods (Qaim, 2020; Beumer and Swart, 2021).

## Conclusion and way forward

To achieve sustainable increase in the rate of genetic gains in food crops, transformative strategies for accelerated crop breeding pipelines need to be embraced. Several national agricultural research system (NARS) initiatives are ongoing under several major initiatives for Africa including Modernizing Ethiopian Research on Crop Improvement (MERCI). Accelerated Varietal Improvement and Seed Delivery of Legumes and Cereals in Africa (AVISA) (https://www.avisaproject.org/), Excellence in Breeding (EiB) Platform (https://excellenceinbreeding.org/), Crops to End Hunger (CtEH) (https://www.cgiar.org/excellence-breeding-platform/crops-to-end-hunger/) and various CGIAR Agri-food research programs (https://www.cgiar.org/research/research-portfolio/). These initiatives put major emphasis on modernizing breeding mainly through developing specific regional product profiles, mechanized operations and databases, besides focusing on infra-structure/human capacity development for efficient breeding and seed systems. Nonetheless, crops face intractable problems not easily solved by traditional breeding and hence there is a need for future breakthroughs in global agriculture. These ongoing breeding modernization agendas integrate innovations in advanced breeding tools (ABTs) such as CRISPR/Cas that are increasingly becoming relevant to fill gaps in the pipeline research required to deliver high yielding, nutritious and climate resilient crop varieties as per the regional demands. Integrating the ABTs such as CRISPR, reverse breeding, double haploids etc. in the “modernized crop breeding platforms” will not only provide game changing solutions to some of the most “intractable” traits but may also be used for enhancing the expression of superior alleles and removal of deleterious effect alleles. Furthermore, these tools and methodologies may be deployed to reverse domestication by editing genes related to domestication traits in wild species making superior lines with enhanced stress resistance for crop improvement. However, accomplishing these desired impacts would require having curated crop genotyping data sets integrated with the trait data from various crop germplasm panels to assist the discovery of trait-specific SNPs and haplotypes for further excavation of superior genes/alleles that may be subsequently deployed for gene editing applications. To support these endeavors, adaptive and user-friendly allele mining platforms need to be in place to manage and mine the massive datasets that have been generated by sequencing reference genomes and re-sequencing efforts on hundreds of new accessions and large transcriptome datasets.

CRISPR/Cas technology has made remarkable progress in recent years for its practical applicability for targeted genome editing in plant species including crop plants. However, certain obstacles such as transformation efficiency and off-target mutations still need to be overcome. For underutilized crops that are less researched, *in vitro* regeneration and transformation pose a major challenging obstacle. Moreover, the genotypic effects on plant regeneration and transformation can be very challenging. To overcome the problems posed by tissue culture and low transformation efficiencies in important crop species, several plant transformation systems such as RNP based systems or transformation free systems need to be established to increase the precision and editing efficiency of plant genome editing.

There have been continuous efforts in development of the tools and applications which has helped us discover newer technologies with each passing decade. The addition of these advanced tools and technologies in the breeder’s tool kit holds tremendous potential to bring changes precisely and efficiently in the genetic makeup of the ruling elite varieties, significantly reducing the need for long breeding cycles for incremental traits speeding up the rate of genetic gains. In addition to the CRISPR/Cas system, several other recently developed systems such as base editing and prime editing have revolutionized the conventional breeding approaches and provided a new direction to the crop improvement programs. With the advancement in new prediction system for on-target activity such as sgRNA CNN (Niu et al., 2021), an array of wider application range has opened leading to an increase in efficiency of crop gene editing and crop improvement programs.

In conclusion, the evolution of genome editing tool kit over the decade has been escalating since the discovery of Cas9 from *Archaea* and undoubtedly, has emerged as the most powerful technology due to its precision, cost effectiveness, and uniqueness to overcome the shortcomings of crop breeding. While ensuing climate change, exploration and creation of additional genetic diversity of underutilized crops require using these precision genetic tools, to create impact on ground, such efforts need to be framed within a breeding pipeline mindset and should be included in the product design process. Although the regulatory pathway for gene edited products is expected to be less complex than for GMOs in several geographies, issues such as freedom to operate and securing the social license need consideration at the intervention design stage. Although CRISPR has potential to deliver disruptive innovations, the trait prioritization should consider the breeding product profiles and market segments for designing and accelerated delivery of locally adapted and preferred crop varieties for the drylands.
